# Leveraging SOLOv2 model to detect heat stress of poultry in complex environments

**DOI:** 10.3389/fvets.2022.1062559

**Published:** 2023-01-06

**Authors:** Zhenwei Yu, Li Liu, Hongchao Jiao, Jingjing Chen, Zheqi Chen, Zhanhua Song, Hai Lin, Fuyang Tian

**Affiliations:** ^1^College of Mechanical and Electronic Engineering, Shandong Agricultural University, Taian, China; ^2^Shandong Provincial Key Laboratory of Animal Biotechnology and Disease Control and Prevention, Shandong Agricultural University, Taian, China; ^3^School of Economics and Social Welfare, Zhejiang Shuren University, Hangzhou, China; ^4^Department of Animal Science and Technology, Shandong Agricultural University, Taian, China; ^5^Department of Digital Urban Governance, Zhejiang University City College, Hangzhou, China

**Keywords:** heat stress, image segmentation, poultry, SOLOv2, biotechnology

## Abstract

Heat stress is one of the most important environmental stressors facing poultry production. The presence of heat stress will reduce the antioxidant capacity and immunity of poultry, thereby seriously affecting the health and performance of poultry. The paper proposes an improved FPN-DenseNet-SOLO model for poultry heat stress state detection. The model uses Efficient Channel Attention (ECA) and DropBlock regularization to optimize the DenseNet-169 network to enhance the extraction of poultry heat stress features and suppress the extraction of invalid background features. The model takes the SOLOv2 model as the main frame, and uses the optimized DenseNet-169 as the backbone network to integrate the Feature Pyramid Network to detect and segment instances on the semantic branch and mask branch. In the validation phase, the performance of FPN-DenseNet-SOLO was tested with a test set consisting of 12,740 images of poultry heat stress and normal state, and it was compared with commonly used object detection models (Mask R CNN, Faster RCNN and SOLOv2 model). The results showed that when the DenseNet-169 network lacked the ECA module and the DropBlock regularization module, the original model recognition accuracy was 0.884; when the ECA module was introduced, the model's recognition accuracy improved to 0.919. Not only that, the recall, AP0.5, AP0.75 and mean average precision of the FPN-DenseNet-SOLO model on the test set were all higher than other networks. The recall is 0.954, which is 15, 8.8, and 4.2% higher than the recall of Mask R CNN, Faster R CNN and SOLOv2, respectively. Therefore, the study can achieve accurate segmentation of poultry under normal and heat stress conditions, and provide technical support for the precise breeding of poultry.

## 1. Introduction

With the continuous growth of the world's population, people have higher requirements for agricultural production. At present, under the extensive and intensive production system, there are more than 3.535 billion animals raised under this system in the world, and the annual output of milk and meat reached 798 million tons and 3,029 million tons respectively (1, 2). According to existing data, it is predicted that global meat consumption will increase by 70% until 2050 (3). Intensified farming has obviously become the main production method to ensure sufficient supply of meat, eggs, and milk from all over the world. But from another perspective, there are many stressors in intensified farming, such as restraint, warmth, density, and immunity (4, 5). Compared with natural grazing and free-range breeding, heat stress (HS) is an important aspect of all stressors, which causes greater harm to poultry production activities.

HS refers to a series of non-specific reactions produced by the body under high temperature stimulation that exceeds the upper limit of the isothermal zone (6). Constant temperature animals have their own isothermal zone, within this range, the animal body can rely on its own physical regulation function to maintain the balance of body temperature, so as to obtain the best body metabolism and physiological functions (7, 8). However, when the temperature exceeds the upper limit temperature of the isothermal zone, the animal's heat dissipation capacity is hindered, what will break the body temperature balance and steady state maintained by the body's own physical regulation, leading to the heat accumulation in the animal's body, resulting in increased body temperature and increased metabolism, which will damage the health and productivity of the animal. And if the animal continues to be in a state of heat stress, symptoms of organ failure will appear, which can lead to animal death if it is not found and treated in a timely and effective manner (9).

Compared with other animals, poultry is more sensitive to high temperature environment. Due to the high body temperature of the poultry itself, rapid metabolism and lack of sweat glands, especially in summer (10, 11), when the poultry is in a high temperature and high humidity environment, the heat loss through evaporation and heat dissipation is reduced, which intensifies the harm of HS. Therefore, Under the general trend of intensive and large-scale development of poultry breeding, how to reduce the impact of HS has become one of the key issues in the process of poultry breeding.

HS brings significant negative impact on the immune system of poultry. For example, it can affect the immunity organs and cytokines of the poultry and do harm to humoral immunity mediated by B cells and T cells. On the other hand, Heat stress can transform the kind of specific immunity of poultry from cell-mediated immunity to humoral immunity, which will increase the susceptibility of poultry to pathogens. Heat stress can also weaken the immune function of poultry against emerging pathogens, so that the number of antibodies after vaccination cannot meet expectations. Therefore, this study designed a poultry heat stress detection model based on machine vision. The model can accurately segment poultry under heat stress from healthy ones, and establish a classifier of heat state. If the system based on this model applied in an actual farm, it can quickly provide heat stress detection results for the breeders, and then provide technical support for the accurate breeding and welfare breeding of poultry.

Traditionally, the temperature-humidity index (THI) is used to determine whether poultry is under HS (12). In general, a THI value of 21 is considered as the threshold for chicken heat stress. However, in the actual breeding process, the temperature and humidity in the poultry house are quite different in space. As an indirect indicator, THI cannot directly reflect the heat stress state of poultry located in different positions of the poultry house. With the rapid development of computer vision and bioacoustic technology, poultry behavior monitoring methods based on images and sounds have been widely studied and applied, thus breaking through the limitations of traditional monitoring methods (13–15). Aydin et al. (16) used 3D images captured by the Kinect depth camera to identify the lying and standing states of broilers, and to detect the number and duration of lying states. According to the negative correlation between lying time and health status of broilers, an indirect method for evaluating the health status of broiler legs was proposed. Pu et al. (17) proposed an automatic convolutional neural network-based method to recognize the chicken behavior within a poultry farm using a Kinect sensor. Compared with the actual results, this test result achieved 99.17% accuracy. In addition, there are also studies on the monitoring of heat stress in poultry. Du et al. (18) concentrated on building a machine learning-based hen vocalization identification algorithm to estimate their thermal comfort status. Using the support vector machine (SVM) algorithm to build a classifier for heat stress state. Data from several studies suggest that the classification performance of the optimal SVM model was 95.1 4.3% (the sensitivity parameter) and 97.6 1.9% (the precision parameter). The typical symptoms of heat stress in chickens are reduced activity and increased drinking time. Therefore, Lin et al. (19) analyzed the changes in exercise and drinking time of chickens under different THI values, and proposed to use time-lapse images and deep learning algorithms to monitor drinking time and coordinates of chickens. Further analysis showed that the detection accuracy rate of this method for chickens was 98.16%, and the tracking accuracy rate for chickens was 98.94%. In conclusion, the analysis of poultry sound and the monitoring of drinking time can accurately determine the heat stress state of poultry. However, these research methods have many shortcomings. For example, it is impossible to accurately locate the position of poultry under heat stress, and long-term monitoring will affect the normal physiological activities of poultry, which is not suitable for actual production.

In order to make up for the insufficiency of existing research, for the images of the complex breeding background in actual production. Based on the existing research, this study improves the DenseNet-169 network and fuses it with the Feature Pyramid Network (FPN) as the backbone network of the SOLOv2 network. A novel classification model (FPN-DenseNet-SOLO) for identifying heat stress status in poultry is proposed. The main contributions of this study are as follows:

(1) For complex background images, a deep learning-based recognition and segmentation model of poultry heat stress state is proposed. The model can complete the accurate segmentation of poultry in normal state and heat stress state, and provides a basis for analyzing the classification model of poultry breeding state.
(2) Optimize the DenseNet-169 network. By introducing Efficient Channel Attention and DropBlock regularization, the extraction of poultry heat stress features is strengthened, and the extraction of invalid background features is suppressed, thereby improving the recognition accuracy and the generalization ability of the network.
(3) Taking the SOLOv2 model as the main framework, the optimized DenseNet-169 is used as the backbone network to fuse the FPN, and instances are detected and segmented on the semantic branch and the mask branch. In this way, the model can solve the problem of gradient disappearance, have strong anti-fitting ability, improve the accuracy of the model, and provide technical support for the monitoring of poultry heat stress state in actual production.

The rest of the paper is organized as follows: Section 2 introduces and summarizes the related work. Section 3 describes the detailed information of the poultry heat stress data set, PoultryHS. Section 4 describes the proposed novel detection model for poultry heat stress in detail. Section 5 provides the evaluation and analyses of the experiment performance. Finally, Section 6 summarizes this paper.

## 2. Materials and methods

### 2.1. Experiment design and image acquisition

**Figure 1:**
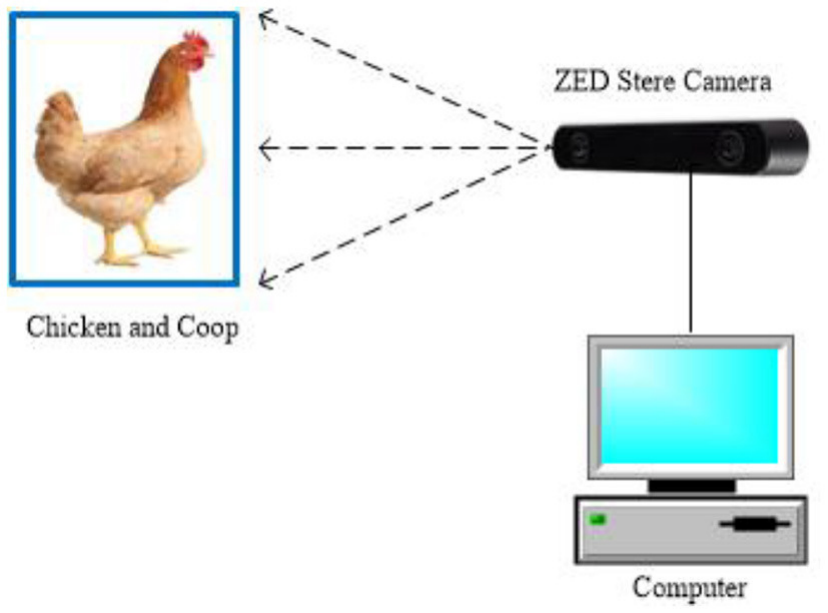
Experiment setup and image acquisition system.

Experiments were conducted between September 10–13, 2021 at Animal Experiment Station of College of Animal Science and Technology, Shandong Agricultural University, Tai'an, Shandong, China. Twenty 13-week-old Hy-Line brown laying hens were randomly selected from the farm and reared. These laying hens are housed in environmentally controlled rooms. Each laying hen is kept in a separate cage, which is connected to each other. Video recording was performed using three ZED 2 cameras to completely record the behavior of the laying hens in the ring control room. It is worth noting that human interference should be avoided during video acquisition. The ZED 2 camera was pre-installed in an environmental control room, the ZED camera was positioned 2.0 m above the ground and precisely at the center of the operation corridor, as shown in Figure 1. The ZED camera was connected to an Intel core i7-11800H CPU, 4.6 GHz, 16 GB physical memory, Microsoft Windows 10 PC installed with the ZED for Windows Software Development Kit (SDK) *via* a USB port. Additionally, the surveillance camera was used to acquire videos (for observation, labeling, and verification) at 30 fps MOV format. The temperature of the environmental control room was set to 27°C and the relative humidity to 60% at the beginning of the experiment. Raised in this environment for 48 h to ensure that the research subjects fully adapt to the new environment. After 48 h, the data collection of laying hens in normal state was carried out. After data acquisition was completed, the temperature of the environmental control room was set to 36°C and the relative humidity was 75%. When the experimental environment reached the set parameters, the image data of laying hens under heat stress were collected. According to the experimental study by Aydin and Mortensen, normal feeding was performed during the experiment, with free access to food and water, and received 16 h of light per day, the light periods having an approximate light intensity of 30 lx.

### 2.2. Definition of heat stress behavior of poultry in images

**Figure 2:**
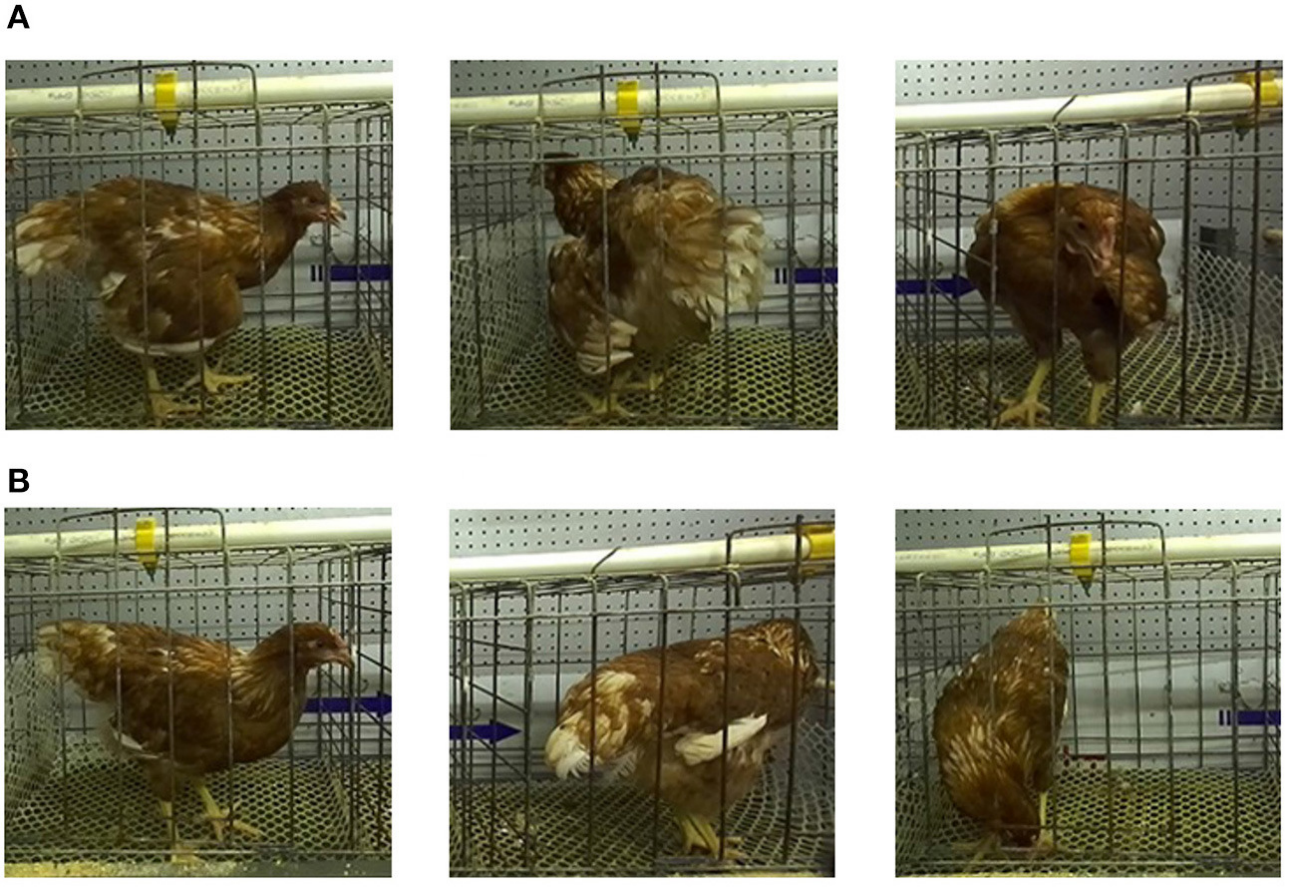
Poultry in different states in the dataset. **(A)** Poultry in heat stress. **(B)** Poultry in normal state.

The breeding environment has an important impact on the growth and production of animals. In the face of different stresses, animals usually exhibit different behavioral characteristics (20, 21). Under high temperature conditions, poultry change their behavioral and physiological balance to thermoregulate and thus lower their body temperatures. And under the influence of heat stress conditions, poultry usually show behaviors such as wing opening, nervous system disorders, short breathing, panting, wing drooping, increased water intake, and reduced food intake (6). As shown in Figure 2A, it can be noticed that the poultry that developed heat stress in this experiment presented behaviors such as wing droop, wing spread, and open mouth panting. These behaviors can be clearly observed in the images compared to the poultry in the normal state (Figure 2B). Behaviors such as wing droop, wing spread, and open mouth panting can increase heat dissipation and thus ensure their own heat stability. This can be seen as a direct behavior of heat stress. At the same time, chickens will drink more water and peck less, which are indirect behavioral effects of heat stress on chickens.

### 2.3. Image pre-processing

A total of 12,741 clear original images were collected by the image acquisition device, and a data set of the poultry heat stress (PoultryHS) was constructed for the first time. The size of these acquired raw images is 2,976 × 2,976 pixels. First, resized the original image to 512 × 512 pixels to facilitate model training. Then, Labelme software was used to label the images of laying hens to generate a mask map. To draw the bounding boxes, we followed the guidelines of the reference Pascal VOC 2010 Challenge (22). Two classification labels are defined: (1) Normal: laying hens under normal conditions; (2) Heat: laying hens under heat stress. As described above, when the poultry were in a suitable environment (temperature of 27°C and relative humidity of 60%), the collected behaviors belonged to the “Normal” label; when the poultry were in a hot environment (temperature of 36°C and relative humidity of 75%), they showed heat stress behavioral characteristics belonged to the “Heat” label. The accuracy of the study was assessed against manually labeled images. The dataset is randomly shuffled and divided into training, test, and validation sets in a 7:2:1 ratio.

## 3. Methodology

Driven by artificial intelligence technology, convolutional neural networks (CNNs) have also achieved rapid development in computer vision. Image recognition and classification technology based on CNNs can realize automatic recognition and classification of target objects; by training a large number of data sets, computers can process, analyze and recognize images efficiently and accurately, which greatly improves production efficiency (23, 24). In the intensive and large-scale animal husbandry mode, there are often some abnormal behavior monitoring problems of livestock and poultry, such as stress response, disease monitoring, behavior recognition, etc. Using CNNs to accurately monitor abnormal behavior of poultry can effectively solve such problems. It is of great significance to improve the economic benefits and management level of the poultry breeding industry.

In order to achieve accurate and rapid detection of the heat stress state of laying hens, this paper proposed to introduce Efficient Channel Attention (ECA) after each convolutional layer on the basis of DenseNet-169 network, and randomly hidden some feature blocks of the research object through DropBlock regularization. In this way, not only the generalization ability of the model can be improved, but also the adaptability of the model to identify poultry in different poses can be enhanced. The improved DenseNet-169 network is used as the SOLOv2 backbone network to fuse the Feature Pyramid Network (FPN) to detect and segment instances on the semantic branch and mask branch. This research will provide technical support for the fine breeding of poultry and animal welfare.

### 3.1. SOLOv2

SOLOv2 is mainly composed of five parts: fully convolutional networks (FCN), feature pyramid network, mask kernel branch, mask feature branch and semantic branch (Category branch). For the input image, the SOLOv2 network divides it into S × S grids, and performs feature extraction through the fully convolutional network and the feature pyramid network to determine whether the center of the instance falls into a certain grid. The grids that meet the conditions will enter the semantic branch and the mask branch, and use the corresponding instances to judge the semantics and the size and position of the mask, respectively (25).

The output of the network is divided into two branches: the classification branch and the semantic branch. The structure of the classification branch is S × S × C, where C is the number of categories. While, the semantic branch structure is H × W × S^2^, where H and We are the high and wide resolution of the semantic output, generally 1/4 of the original image, and S^2^ is the maximum number of instances predicted. One-to-one correspondence with the grid of classification branches in a top-to-bottom, left-to-right manner. According to the principle, the network predicts category and semantic segmentation based on the location at the same time, and combines the results to achieve instance segmentation. Semantic prediction is divided into convolution kernel branch and feature branch. Matrix non-maximum suppression is proposed in SOLOv2. The idea is derived from softening non-maximum suppression, but the computational efficiency is close to that of fast non-maximum suppression. SOLOv2 has a simple structure, and the one-step instance segmentation method adopted has high efficiency and accuracy.

### 3.2. DenseNet

The SOLOv2 network has a complex structure and generates a high number of parameters. In order to reduce the number of parameters and improve the model performance, the DenseNet-169 network is selected as the backbone network in this study. The robust structure of dense block in DenseNet network makes the number of output feature maps of each convolutional layer small. Compared with traditional convolutional networks, it requires fewer parameters to make the feature transfer more efficient, make the network easier to train, make the number of parameters smaller, and make the performance improvement higher. At the same time, the model uses the feature maps of all previous layers as input and its own feature maps as input to all subsequent layers, ensuring maximum information transfer between layers in the network. In this way, all layers can be connected and the gradient disappearance can be effectively mitigated. The DenseNet network combines the advantages of Reset and Inception, but simply improves the performance of the DenseNet network by combining them, but directly from the perspective of optimal features. By using the two structures of feature reuse and bypass setting, the amount of parameters of the network can be reduced, and the problem of gradient disappearance can be alleviated (26, 27).

**Figure 3:**
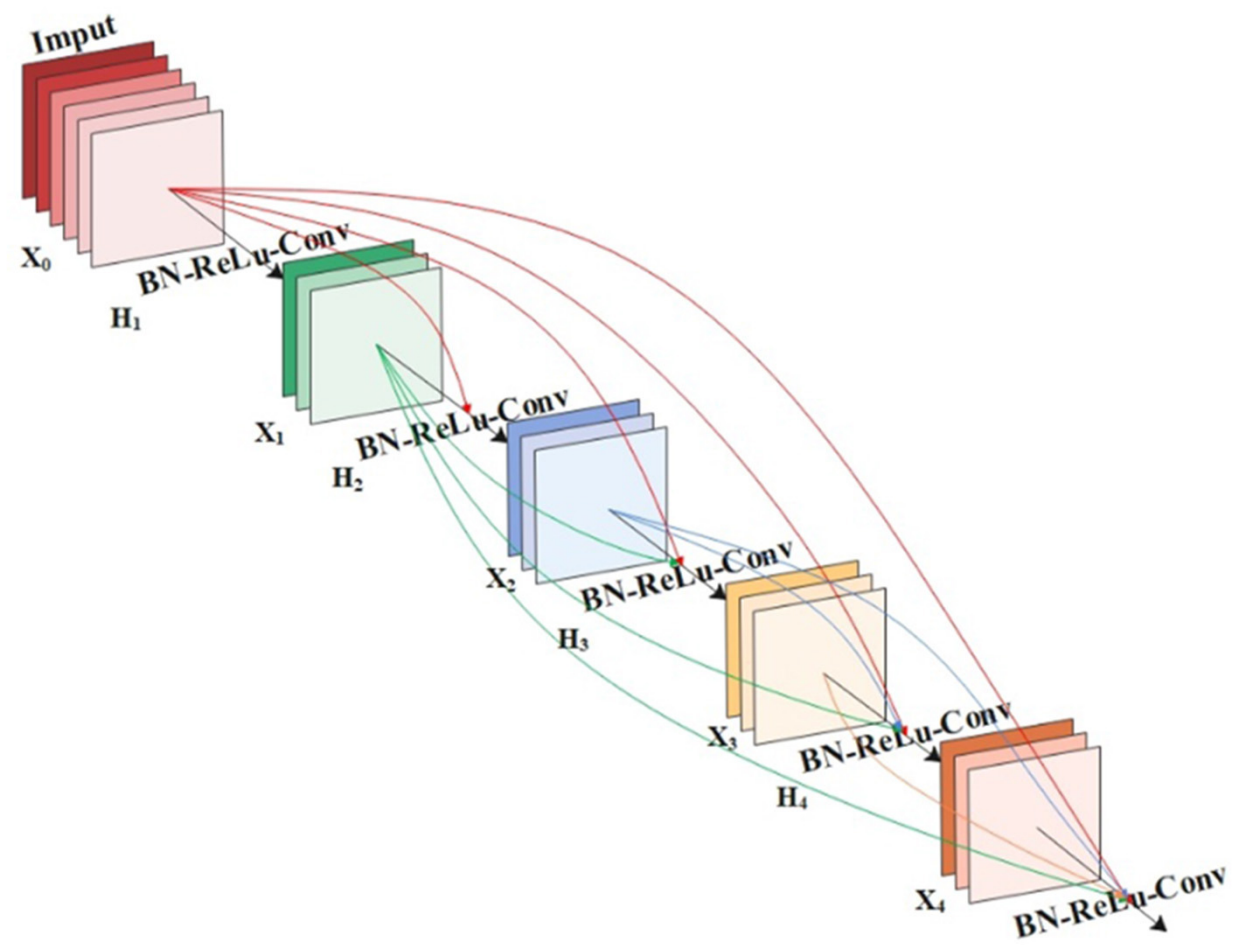
Layer Denseblock network structure.

DenseNet is a densely connected convolutional neural network, mainly composed of DenseBlock and Transition Layer. The architecture contains convolutional layers, pooling layers, and densely connected modules with a growth rate of 4, where the growth rate is to keep the channel feature dimension moderate. As shown in Figure 3, DenseNet contains multiple DenseBlock modules, DenseBlock consists of BN + ReLU + Conv (l × 1) + BN + ReLU + Conv (3 × 3), and the layers between DenseBlocks are called Transition Layers, which consist of BN + Conv (l × 1) + Average Pooling (2 × 2). Since the dimension of the output feature map is too large, an l × 1 convolution is added to the Transition Layers module for dimensionality reduction, thereby improving computational efficiency.

In the DenseNet network, there is an inseparable relationship between any two layers, that is, the input of any layer in the network is the superposition of the output results of all previous layers. The result obtained by this layer will also be transmitted to the next layer as input with the previous output, which is transmitted down in turn. Use the feature outputs of all previous layers as the input of the current layer, that is, X_0_, X_1_, …X_l−1_ are the feature maps from the first layer to the l-1 layer, pass through the l-th layer through the cascade connection, and finally use the composite function H_l_ (^*^) to obtain the output X_l_.


(1) $$\begin{array}{l} X_{l}=H_{l}\left(\left[X_{0},X_{1},...,X_{l-1}\right]\right) \end{array}$$

Where H_i_ is BN + ReLU + 1 × 1 Conv + BN + ReLU + 3 × 3 Conv; BN means Batch Normalization; ReLU is the activation function; Conv is the convolution; [X_0_, X_1_,…, X_i−1_] means splicing all the feature layers before the i-th layer.

### 3.3. Efficient Channel Attention (ECA-Net)

**Figure 4:**
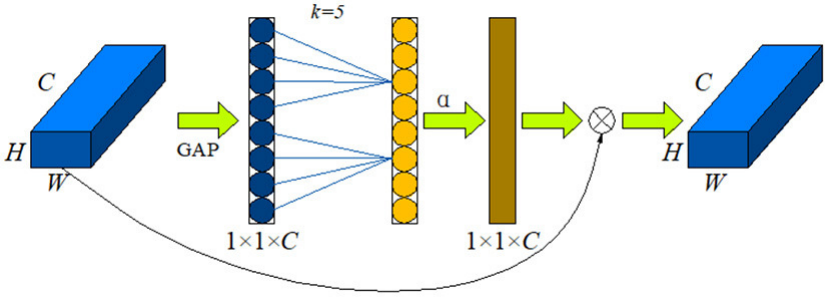
ECA module structure diagram.

Chicken cages, troughs, water troughs and other poultry co-exist within the feeding area are the backgrounds in the actual production environment, which are complex and diverse. Based on the backbone network, the efficiency of extracting behavioral features from images is improved by adding an efficient attention mechanism to increase the weights of important features, thus strengthening the poultry heat stress behavioral features and suppressing the invalid background features. This study adopts ECA-Net, a lightweight attention module that can improve the performance of deep convolutional neural networks. By using the efficient attention module to combine the depth and spatial information of the feature map, the extraction of important features can be effectively suppressed while the extraction of non-important features can be effectively suppressed, so that the accuracy of target recognition in complex environments can be effectively improved (28). Figure 4 is the structure diagram of ECA-Net. C is the number of channels, H is the height of the input data, W is the width of the input data, k is the local interaction size of one-dimensional convolution, GAP is the global average pooling, σ is the sigmoid activation function. The same below. Firstly, global average pooling was performed on the input feature map, and a single value was used to represent the feature layer of each channel; Second, used a one-dimensional convolution of size k to generate weights for each channel, obtained the interdependence between each channel, and added a sigmoid activation function for normalization; Finally, the weights generated by each channel were multiplied onto the input feature map to enhance the extraction of important features.

ECA-Net uses a one-dimensional convolution of size k to interact across channels to replace the fully connected layer, which can effectively reduce the computational complexity and complexity of the fully connected layer, and then generate weights for each channel.


(2) $$\begin{array}{l} \omega =\delta \left(CID_{k}\left(y\right)\right) \end{array}$$

where ω is the channel weight, δ is the sigmoid activation function, and CID is one-dimensional convolution. The more channels of the input feature map, the greater the k value that requires local interaction, so the k value is proportional to the number of channels C. In this paper, the k value is determined adaptively by a function related to the channel dimension.


(3) $$\begin{array}{l} C=2^{\left(\gamma \cdot k-b\right)} \end{array}$$

Therefore:


(4) $$\begin{array}{l} k={\left|\frac{\log_{2}\left(C\right)}{\gamma }+\frac{b}{\gamma }\right|}_{odd} \end{array}$$

Where |*t*|_*odd*_ is the odd number closest to t, γ, and b is set to 2 and 1, respectively.

### 3.4. DropBlock regularization

Changes in the surrounding environment may lead to a reduction in the recognition accuracy and the over-fitting of the DenseNet-121 network. In this paper, the DropBlock regularization model was adopted to avoid over-fitting by randomly hiding some feature maps, so as to extract higher robust features.

DropBlock is an improved version of Dropout, which can remove semantic information more effectively than Dropout (29, 30). DropBlock works on the entire space block. Dropout regularization generally works by randomly hiding neurons in fully connected layers (31), however, it is not very effective when used in convolutional layers. The reason is that with the deepening of feature extraction, the feature map gradually becomes smaller, and the receptive field gradually becomes larger. Each feature on the feature map corresponds to a receptive field range, and the corresponding semantic information can be learned through adjacent position elements, and then loses its effect. On the other hand, DropBlock hides the feature map by setting the whole block element, blocks the learning semantic information of adjacent positions, and normalizes the feature map that is not hidden, so as to achieve the regularization effect of the convolution layer.

s and γ are two important parameters in DropBlock: s represents the size of the hidden block. Generally, the network takes 3, 5, and 7, and the effect is best when s = 7; γ represents the number of hidden activation units. The relationship between them is shown in Eq. (5).


(5) $$\begin{array}{l} \gamma =\frac{1-\rho }{s^{2}}\cdot \frac{f_{s}^{2}}{\left(f_{s}-s+1\right)} \end{array}$$

Where ρ represents the activity threshold probability of the activation unit, and *f*_*s*_ represents the size of the feature map there.

### 3.5. FPN-DenseNet-SOLO model

**Figure 5:**
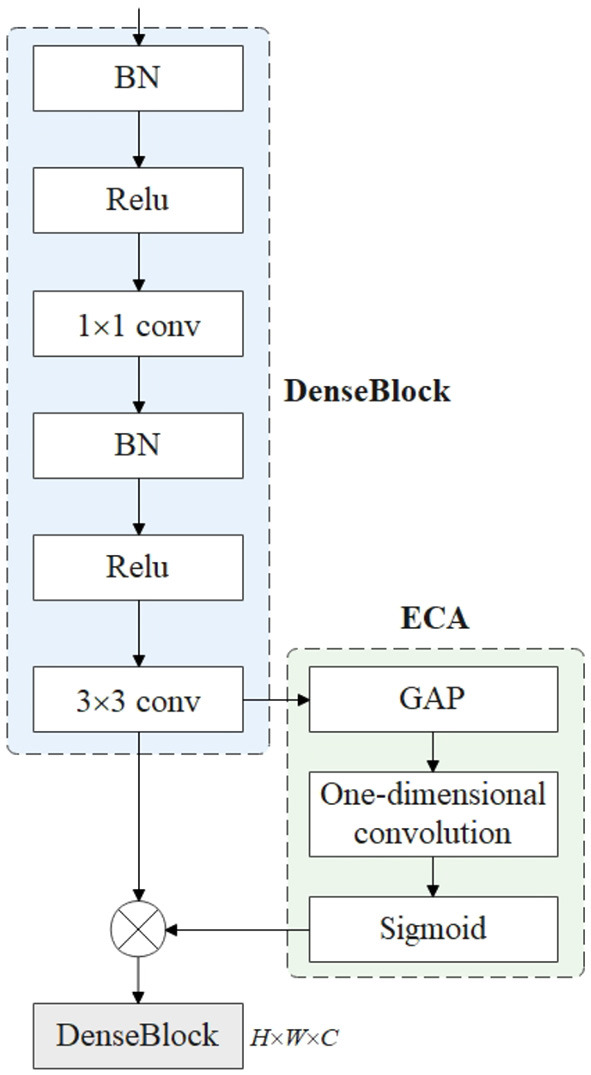
ECA-DenseBlock structure diagram.

The DenseNet-169 network is mainly composed of DenseBlock and Transition Layer. The number and width of output feature maps for each convolutional layer in DenseBlock are lower than the input feature maps. This form of connection makes the transfer of features and gradients more efficient. Figure 5 is a structural diagram of ECA-DenseBlock. DenseBlocks are connected to each other through operations such as BN-ReLU-conv. The ECA-Net module is added to each DenseBlock module to calibrate the weight of each channel on the feature map, increase the weight of important features, and suppress useless features, thereby strengthening the poultry features in heat stress state, while suppressing complex poultry house background features. The ECA-DenseBlock network can effectively speed up the training speed, improve the accuracy of the model, and effectively improve the performance of the network. Since each feature layer contains the output information of the previous feature layer, the information flow is enhanced, so only a few feature maps are needed to complete the network training.

**Figure 6:**
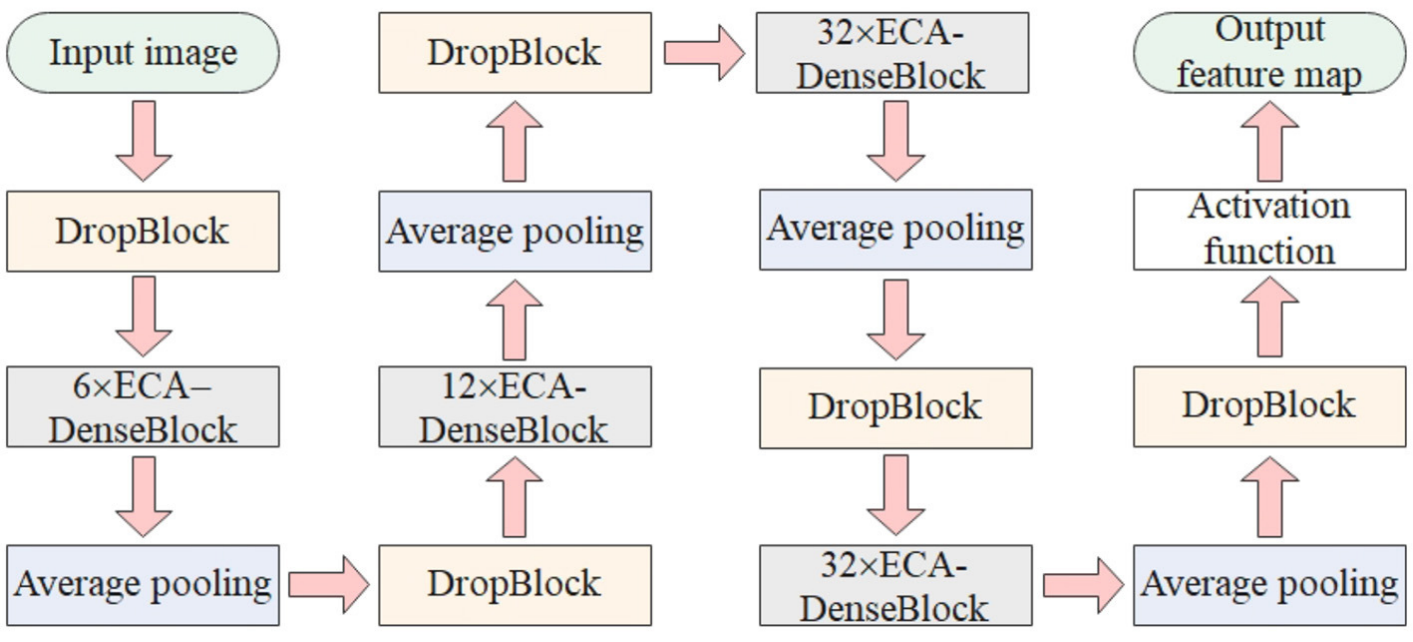
Improved DenseNet-169 network structure.

Figure 6 shows the improved DenseNet-169 network structure. First, the input color image goes through a convolutional layer with a 7 × 7 convolution kernel, which adjusts the number of channels of the image and extracts effective information. Then connect a DropBlock regularization module and an ECA-DenseBlock module in turn. The DropBlock module hides the feature map by setting the whole block element, blocks the learning semantic information of adjacent positions, and normalizes the feature map that is not hidden, so as to realize the regularization of the convolution layer and improve the generalization ability of the model. ECA-DenseBlock is the core part of the improved model. The improved network contains a total of 4 ECA-DenseBlock modules (the number of dense connections is 6, 12, 32, 32). A Transition Layer is connected after each ECA-DenseBlock. Among them, 1 × 1 conv and average pooling are used to adjust the number of channels to prevent the feature dimension from growing too fast. After the features are extracted by the dense connection structure with the addition of attention mechanism, DropBlock regularization is added to prevent overfitting. Using global average pooling and sigmoid activation function, the generated weights of each channel are weighted onto the feature map by multiplication, and finally the processed feature map is output.

The original SOLOv2 network mainly consists of 5 parts: fully convolutional networks (FCN), feature pyramid network (FPN), mask kernel branch, mask feature branch and category branch. Before the model predicts instance information, it must perform feature extraction through a convolutional network. Compared with FCN, Densenet can fuse the features of each layer of the neural network through dense connections, and the features are reused, which can reduce the amount of calculation and the number of parameters. Its jump structure enables the input feature maps of each layer to be directly connected to the final loss function, accepting the supervision of the final loss function, solving the problem of gradient disappearance, making the network easy to train, and obtaining strong anti-fitting ability.

**Figure 7:**
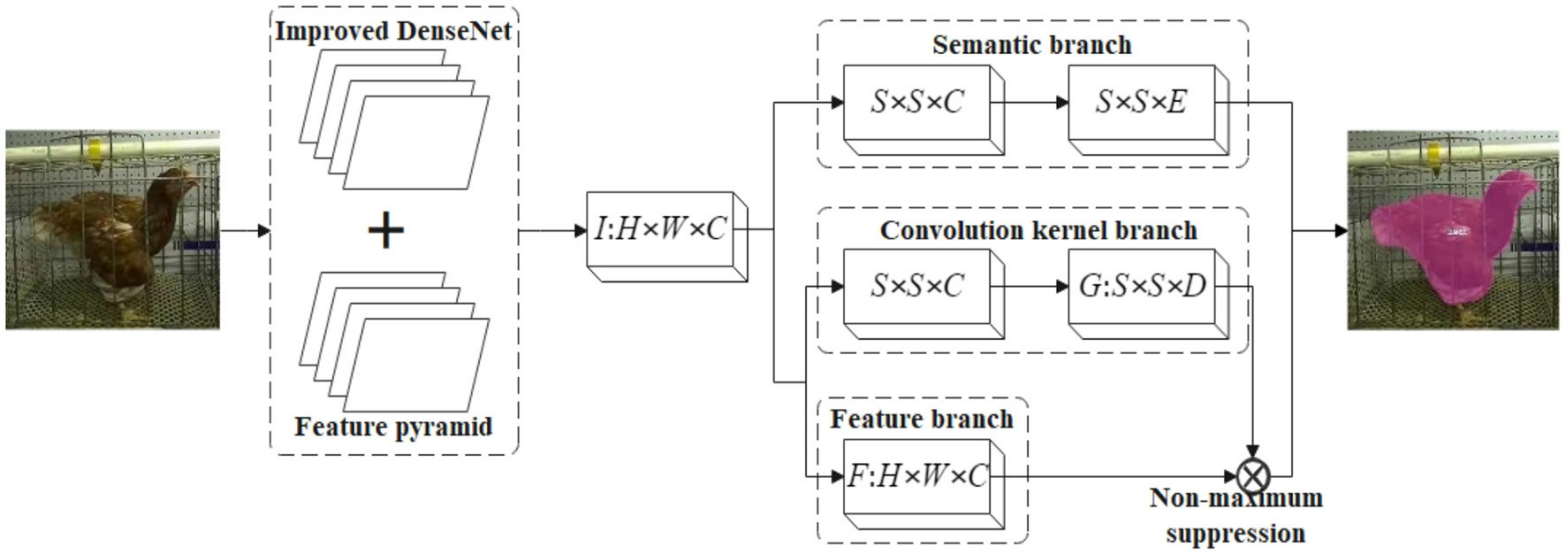
Improved SOLOv2 network structure.

In Figure 7, H, W, and We are the height, width and number of channels of the output feature map I and mask feature map F of the feature pyramid, respectively. S represents the height or width of the feature map after alignment; C is the total number of semantic categories, which is equivalent to the number of channels of the semantic branch output feature map; D is the convolution kernel weight, which is equivalent to the number of channels of the mask kernel G. The input image is divided into S × S grids, the total category is C, and the input space is H × W × E before being sent to the semantic branch through the feature pyramid, and aligned to S × S × E. After semantic branch processing (multiple 3 × 3 convolutions), it is expanded into an output space of S × S × C. It means that S × S C-dimensional outputs are finally generated, and the semantic category probability is predicted for each grid. Predict the size and position of the mask in the mask branch, and take the output feature map I of the feature pyramid as the input. Finally, the mask kernel *GϵR*^S×S×D^ and mask feature map *FϵR*^H×W×E^ are output after two branches of calculation and learning.

## 4. Experimental results and analysis

### 4.1. The setup of experiments

The software environment experimental platform is Ubuntu 18.04 LTS 64-bit system. Python 3.6 is selected for programming language and Pytorch 1.1 is picked up as the deep learning framework equipped with Intel^®^ Xeon(R)CPU E5-2683 V3 processor and NVIDIA GeForce GTX 1080Ti GPU, respectively.

The parameters of the CNNs were set as follows: Adam optimizer was used to optimize the model, and the cross entropy was used as the loss function. The batch size was 64, the number of iterations was 100, the initial learning rate was 0.01, the learning rate was reduced to 1/10 of the initial value if the loss value of the validation set does not decrease after 10 epochs. To minimize randomness during training, set random seeds to 100.

### 4.2. Loss function

The selection of the loss function is one of the important links in the training process of the deep learning network model. Choosing the correct loss function can provide a better convergence direction and obtain better training results. The current general loss function is the L_2_ loss function, and the square of the Euclidean norm of the difference between the minimized prediction result and the true value is used as the convergence direction. The function definition is shown in formula (6):


(6) $$\begin{array}{l} l_{2}\left(\bar{\overset{⌢}{y}}-y\right)=||\bar{\overset{⌢}{y}}-y|{|}_{2}^{2} \end{array}$$

From the formula, it can be found that when the difference between the predicted value and the real value is large, the L_2_ loss function can rapidly decrease the gradient and improve the convergence speed of the model. However, when the predicted value is close to the real value, the convergence speed of the L_2_ loss function will be greatly reduced, and the gradient descent will be slow. Therefore, this paper adopts the BerHu loss function, which can effectively combine the L_1_ loss function with the L_2_ loss function to obtain better convergence. Its definition is shown in formula (7):


(7) $$\begin{array}{l} B\left(x\right)=\{\begin{array}{c} |x||x|\leq c\\ \frac{x^{2}+c^{2}}{2c}|x|>c \end{array} \end{array}$$

The BerHu function takes c as the limit, and works with the L_2_ loss function when it is greater than *c* to ensure that the gradient decreases rapidly; When it is less than *c*, it works with the L_1_ loss function to ensure that when the predicted value is close to the real value, the gradient of the model can also maintain a certain speed of decline. This paper set $c=k\max_{i}(|\bar{\overset{⌢}{y}}|-y)$, and set k to different values (0.1, 0.5, and 0.2) for testing. The test results showed that when k = 0.2, the model obtained the best output results.

### 4.3. Model training

**Figure 8:**
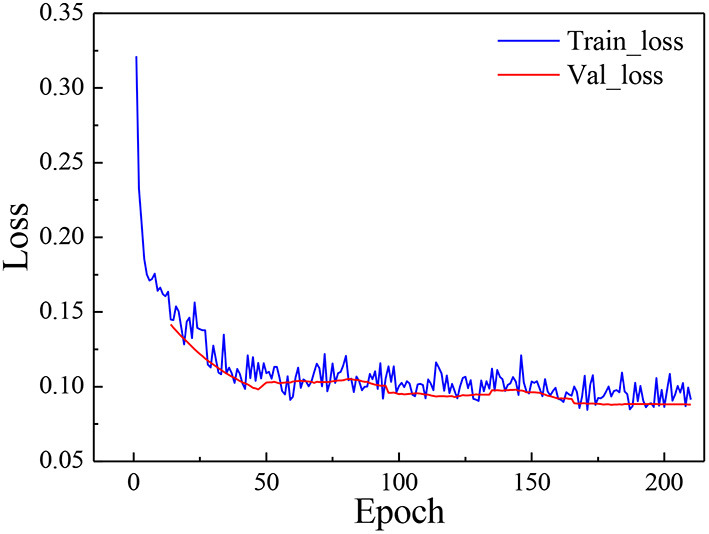
Loss curve of the model.

In the experiment, the poultry heat stress data set (PoultryHS) was used for training. And the training set, validation set and test set were obtained by dividing them according to the ratio of 7:2:1. All three used the same classification, which was convenient to objectively measure the model recognition ability. The loss curves of the model on the training set and validation set were shown in Figure 8.

The loss curve can intuitively reflect the dynamic process of training, and can also reflect the convergence of the network through the change of the loss value. Figure 1 showed the variation trend of the loss function with the number of iterations throughout the training process. After the model completed an epoch training, the parameters of the model were adjusted using the validation set. As the epoch increased, the Loss curve of the model gradually decreased and tended to stabilize. Specifically, during the first 9 epochs, the loss value dropped rapidly. Then between 9 and 40 epochs, the rate of decline in the loss value flattened out. Between 40 and 165 epochs, there was a small fluctuation in the loss value. After 165 epochs, the fluctuation of the loss value was further reduced, and the overall value had become stable, which meant that the network convergence effect was good. At this point the training loss was 0.0871 and the validation loss was 0.0881.

### 4.4. Detection effect

**Figure 9:**
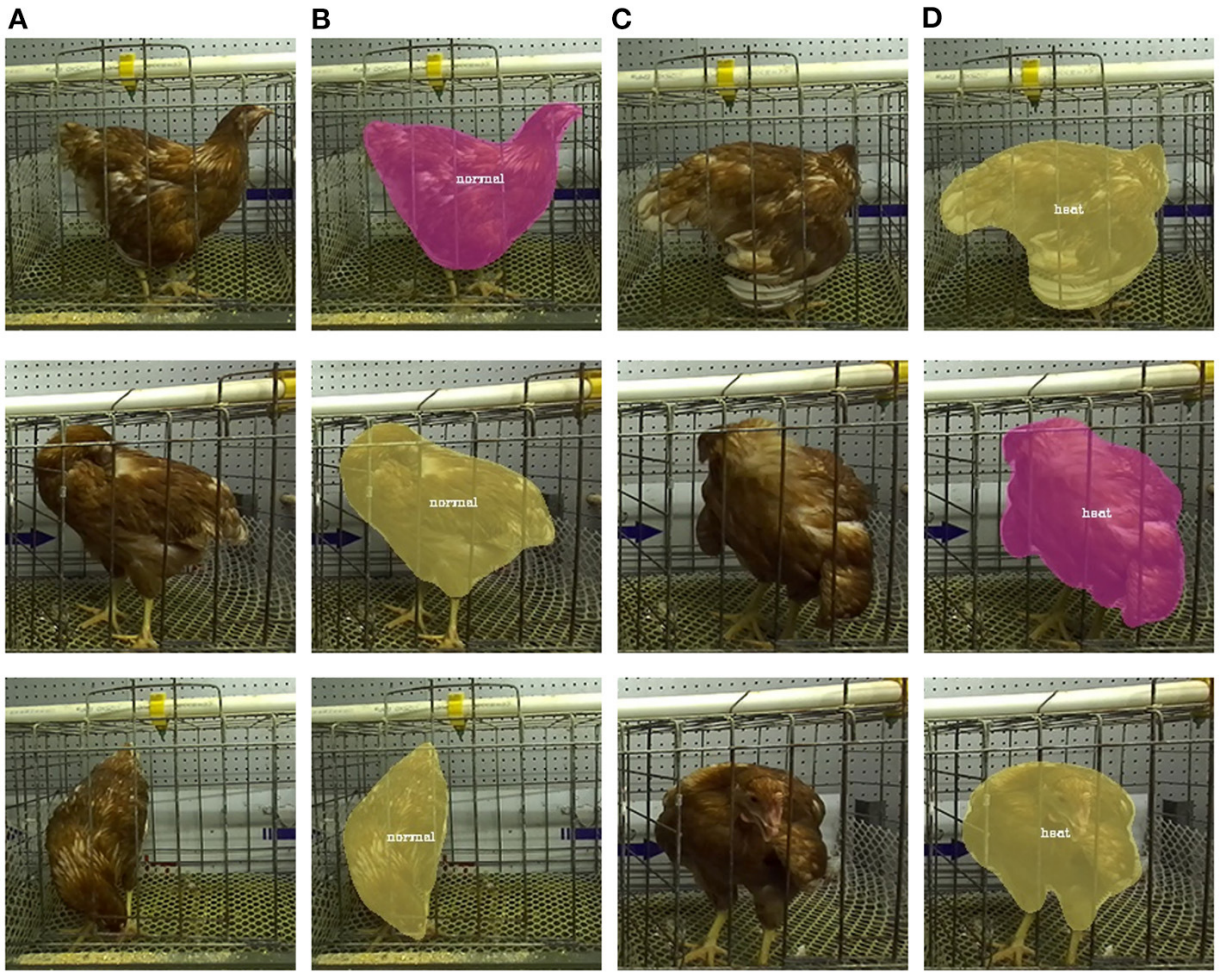
Model segmentation effect. **(A)** Laying hens in normal state; **(B)** identification results; **(C)** laying hens in heat stress state; **(D)** identification results.

Since the farm environment in which the dataset was collected was a non-open-air poultry house, external weather factors had little influence on the test results. And the test did not consider the situation of lights out at night, so there is no night-time detection in the recognition results. As can be seen from Figure 9, the FPN-DenseNet-SOLO model had a good detection effect, not only could accurately segment hens from complex environments, but also could accurately identify the posture of hens. According to the different postures and standing angles of hens, the algorithm could show high accuracy and recognition rate, and could also effectively recognize hens with less obvious characteristics.

### 4.5. Comparison of identification results with classical CNN models

To further analyze the performance of the FPN-DenseNet-SOLO model, the classic CNNs models (Mask R CNN, Faster R CNN, SOLOv2) are compared with the improved models under the same experimental conditions. Use the same training set, validation set and test set to train and test different CNNs models. Table 1 showed the comparison of the classification results of different CNNs models on the test set for poultry heat stress state. In the table, AP_0.5_ and AP_0.75_ represented the AP indicators when the IoU threshold was 0.5 and 0.75, respectively; mAP represented the average of AP corresponding to the increase of the IoU threshold from 0.5 to 0.95 in steps of 0.05.

**Table 1:** Recognition results of different CNNs models.

**Model**	**Recall**	**AP_0.5_**	**AP_0.75_**	**mAP**	**Testing time (s)**
Mask R CNN	0.804	0.828	0.539	0.641	0.215
Faster R CNN	0.866	0.873	0.646	0.713	0.179
SOLOv2	0.912	0.936	0.847	0.792	0.078
FPN-DenseNet- SOLO	0.954	0.978	0.934	0.909	0.092

Each model trained to convergence was applied to the test set for instance detection and segmentation of poultry heat stress, and the final performance evaluation results were obtained as shown in Table 1. From the table, it could be found that the recall, AP_0.5_, AP_0.75_ and mAP of FPN-DenseNet-SOLO model on the test set were higher than other networks. The recall of FPN-DenseNet-SOLO was 0.954, which was 15%, 8.8% and 4.2% higher than the recall of Mask R CNN, Faster R CNN and SOLOv2, respectively. Compared with the original SOLOv2 network, the model proposed in this paper outperformed in various average precision (AP_0.5_, AP_0.75_, and mAP) metrics, 0.978, 0.934, and 0.909, respectively. Especially when the IoU threshold was 0.75, the improvement effect was very obvious, reaching 8.7%. In addition to evaluating the average accuracy of FPN-DenseNet-SOLO, it is also necessary to consider the time spent by the algorithm in the actual segmentation, that is, to reduce the segmentation time while ensuring the accuracy. On the same Graphics Processing Unit (GPU), Mask R CNN, Faster R CNN, SOLOv2, and FPN-DenseNet- SOLO models had an average segmentation time of 0.215, 0.179, 0.078, and 0.092 s to recognize an image, respectively. SOLOv2 and improved SOLOv2 were significantly faster in processing than the other two models, and SOLOv2 model had the shortest split time. FPN-DenseNet-SOLO was only 0.014s slower than SOLOv2, the gap was almost negligible. Considering both average precision and Testing time, the performance of FPN-DenseNet-SOLO is better than SOLOv2. The above results show that the improved method of SOLOv2 network is feasible, and the proposed model can effectively detect the heat stress state of poultry, which highlights the superiority of the model in the identification of heat stress in poultry.

### 4.6. Ablation experiment

According to the comparative analysis of the identification and classification results of different CNNs models on the heat stress state of poultry on the test set, the FPN-DenseNet-SOLO model has the best performance. Therefore, this paper extracts the features of the improved model and analyzes the influence of different backbone networks on the detection results. Ablation experiments were performed on the proposed model, and the results are shown in Table 2. When the ECA-Net module and DropBlock regularization module were not added to the DenseNet-169 network, the original model recognition accuracy was 0.884. The introduction of the ECA-Net module increased the recognition accuracy of the model to 0.905. This shows that the depth and spatial information of the feature map are combined, which can efficiently extract image feature information, such as texture, edge and color, and suppress the extraction of new questions in complex environments. As the convolutional layers deepen, the visual information in the feature map will be further reduced, increasing the amount of abstract information. The addition of the ECA-Net module effectively improves the accuracy of the identification and classification of poultry heat stress in complex poultry house environments. The model recognition accuracy after using the DropBlock regularization module is 91.8%. The function object of this module is the entire space block. By randomly hiding some feature blocks of poultry images, some continuous semantic information on the feature map is deleted. In addition, some independent units can also be randomly discarded, but the deletion of relevant semantic information is not complete, and the corresponding feature information can still be passed to the subsequent network layers. Therefore, the addition of DropBlock regularization can improve the generalization ability of the model. Faced with poultry with different postures and feathers, it can effectively enhance the adaptability of model recognition. After the introduction of ECA-Net and DropBlock regularization module improvement, the new model has a recognition accuracy of 0.919 for heat stress in poultry, which is 3.5% higher than the original model. The experimental results show that the addition of ECA-Net and DropBlock regularization module can better extract image features of poultry heat stress.

**Table 2:** Results of ablation experiments.

**ECA-Net**	**DropBlock**	**mAP**	**Recall**
–	–	0.884	0.915
√	–	0.905	0.932
–	√	0.897	0.928
√	√	0.919	0.954

### 4.7. Limitations

Compared with the original SOLOv2 model, the detection performance and detection time of FPN-DenseNet-SOLO have been greatly improved. The model inference speed ensures the feasibility of real-time detection, and accurate judgment can be made even when the complex environmental factors of the poultry house are greatly affected.

Nevertheless, as the FPN-DenseNet- SOLO model was applied to edge computing and cloud computing, the size of the model was large, which was not easy to be used in mobile platforms or embedded development platforms. As a result, the application scope of the model was limited and the degree of portability was not high. In future research, we will focus on reducing the size of the model without losing the accuracy of model recognition, so that it can be applied to mobile platforms and embedded development platforms to make the model more widely applicable.

## 5. Conclusion

To achieve accurate and rapid identification of poultry heat stress state, this paper proposes a poultry heat stress detection algorithm based on FPN-DenseNet-SOLO. The conclusions are as follows.

(1) In the complex background, the recall, AP_0.5_, AP_0.75_ and mean Average Precision of the FPN-DenseNet-SOLO model on the test set were higher than other networks. The recall of this model was 0.954, which was 15, 8.8, and 4.2% higher than the recall of Mask R CNN, Faster R CNN, and SOLOv2, respectively.
(2) DenseNet-169 was optimized by introducing ECA and DropBlock regularization, which strengthened the extraction of poultry heat stress features, improved the recognition accuracy and the generalization ability of the network. Taking the SOLOv2 model as the main framework, the optimized DenseNet-169 was used as the backbone network to fuse FPN, and instances were detected and segmented on the semantic branch and mask branch.
(3) The FPN-DenseNet-SOLO model solved the problem of gradient disappearance, enhanced the anti-fitting ability, improved the accuracy of the model, and provided technical support for monitoring the heat stress state of poultry in actual production.

## Data availability statement

The raw data supporting the conclusions of this article will be made available by the authors, without undue reservation.

## Ethics statement

The animal study was reviewed and approved by Animal Ethical Review Committee of Shandong Agricultural University.

## Author contributions

ZY and FT contributed to the concept and method. LL and JC contributed to the draft and revision of the manuscript. All these authors contributed to the experiments.
